# Green Chemometric-Assisted Characterization of Common and Black Varieties of Celery

**DOI:** 10.3390/molecules28031181

**Published:** 2023-01-25

**Authors:** Alessandra Biancolillo, Martina Foschi, Leila D’Alonzo, Valter Di Cecco, Marco Di Santo, Luciano Di Martino, Angelo Antonio D’Archivio

**Affiliations:** 1 Department of Physical and Chemical Sciences, University of L’Aquila, Via Vetoio, Coppito, 67100 L’Aquila, Italy; 2 Majella Seed Bank-Parco Nazionale della Majella, Via Badia, 28, 67039 Sulmona, Italy

**Keywords:** celery, geographical origin, classification, authentication, class-modeling, discriminant analysis, SPORT, SIMCA

## Abstract

Celery (*Apium graveolens* L., var. Dulce), is a biennial herbaceous plant belonging to the Apiaceae family, cultivated in humid soils in the Mediterranean basin, in Central-Southern Europe, and in Asia. Despite its wide diffusion and although it is well-known that cultivar/origin strongly influences plant composition, only a few studies have been carried out on the different types of celery. The present work aims to investigate four different Italian types of celery (two common, Elne and Magnum celery, and two black, Torricella Peligna Black and Trevi Black celery), and to test, whether the combination of FT-IR spectroscopy and chemometrics allows their ecotype discrimination. The peculiarity of this study lies in the fact that all the analyzed celeries were grown in the same experimental field under the same soil and climate conditions. Consequently, the differences captured by the FT-IR-based tool are mainly imputable to the different ecotypes. In order to achieve this goal, FT-IR profiles were handled by two diverse classifiers: sequential preprocessing through ORThogonalization (SPORT) and soft independent modeling by class analogy (SIMCA). Eventually, the highest classification rate (90%, on an external set of 100 samples) has been achieved by SPORT.

## 1. Introduction

*Apium graveolens* L., var. Dulce, commonly known as celery, is a biennial herbaceous plant belonging to the Apiaceae (or Umbelliferae), family, cultivated in humid soils in the Mediterranean basin, in Central-Southern Europe, and in Asia [1].

Celery provides a low-caloric intake, but it is very rich from a nutritional point of view. In fact, it is a considerable source of water, minerals (such as potassium, magnesium, calcium, phosphorus, iron, sodium, zinc, and copper), fibers, vitamins (mainly A, C, E, K, B1, B2, B6) and antioxidants. On average, concerning macronutrients, it contains 46% of proteins, 45% of carbohydrates, and 9% of lipids [2,3,4].

Despite its wide diffusion in the culinary tradition of different nations, and although it is well-known that cultivar/origin strongly influences plant composition, only a few studies have been carried out on different types of celery. One of these is the work published by Liu and coauthors [5], who analyzed four different celery cultivars (Benqin, Western, Baoqin, and Majiagou) grown in China using high-performance liquid chromatography–mass spectrometry (HPLC–MS) and then compared the antioxidant composition in leaves and petioles. Eventually, they demonstrated that the inter-cultivar differences (in terms of antioxidant composition) were significant. 

Another study, focused on the metabolomic investigation of celeries harvested in different geographical areas, was carried out by Lau et al. [6]. In this work, 1H NMR was combined with principal component analysis (PCA) and partial least squares discriminant analysis (PLS-DA) for the discrimination of extracts obtained from celeries harvested in Australia, Taiwan, and China. The authors concluded that the proposed approach is suitable for tracing celeries and that mannitol, sucrose, citric acid, glutamine, asparagine, and 4-hydroxybenzoic acid can be used as markers of origin.

In 2021, Reale and collaborators [7] investigated the volatile composition of two Italian celery ecotypes: the Sperlonga White and the Torricella Peligna Black, by means of head-space solid-phase microextraction combined with gas-chromatography/mass spectrometry (HS-SPME/GC-MS) to evaluate resemblance/differences among the volatilome of these species and the commercial ones. The volatile compound composition thus obtained (both on the leaves and on the petioles), was then analyzed using an exploratory method. In particular, the data were analyzed by principal component analysis (PCA), in order to evaluate whether there were any trends or similarities/dissimilarities between the aromatic profiles of the different samples. The authors concluded that Torricella Peligna Black celery exhibited a significantly different composition in volatile profile with respect to the others. Furthermore, they demonstrated that the composition of the volatile profile was suitable for discriminating celery petioles according to the harvesting time. Recently, Biancolillo et al. investigated Elne and Torricella Peligna black celeries coupling e-eye and chemometrics [8]. The proposed approaches consist of the analysis of the leaves using a microscope, and the subsequent extraction of the colorgrams from the obtained RGB images. Then, data were analyzed via Sequential preprocessing through orthogonalization linear discriminant analysis (SPORT-LDA), sequential and orthogonalized covariance selection linear discriminant analysis (SO-CovSel-LDA), and a class-modeling method called soft independent modeling of class analogies (SIMCA). The lowest prediction errors (on the external test set) were provided by SPORT-LDA and SO-CovSel-LDA. This approach is particularly interesting because, similarly to the methodology proposed in the present work, it allows to classify different ecotypes of celery by means of a green and completely non-destructive method. Starting from this point, the present work aims to test whether it is possible to develop an FT-IR-based tool for the discrimination of black and common types of Italian celeries. In particular, the comparison has been made among two different ecotypes of black celery, the Torricella Peligna Black and the Trevi Black, and two common cultivars: Elne and Magnum. Finally, the FT-IR profiles have been handled by two classifiers: sequential preprocessing through ORThogonalization (SPORT) and soft independent modeling by class analogy (SIMCA). The choice of using FT-IR spectroscopy is dictated by the nowadays need of developing more sustainable methods, which are possibly faster and require less expensive sample preparation. In fact, the universal Attenuated Total Reflectance (uATR-) FT-IR instrumentation allows a non-destructive or semi-destructive analysis; it does not require sample preparation, and it excludes the need for chemical reagents (except for minimum quantities of methanol used to clean the instrument between measurements). Consequently, the proposed methodology appears as a green alternative to state-of-the-art methods (mainly chromatographic). The two mentioned classifiers have been chosen to test how two different natured approaches would behave on this data set, and because they have outperformed in similar contexts [9,10]. In the case of the discriminant approach, all the classes have been simultaneously investigated. On the other hand, SIMCA models focused on the two local ecotypes of celeries grown in Central Italy. 

The present work aims to demonstrate a different chemical (and, therefore, organoleptic) composition among the different celery landraces, assisting in the recognition of the peculiarities of the local ecotypes and laying the foundations for their protection.

## 2. Results and Discussion

**Figure 1:**
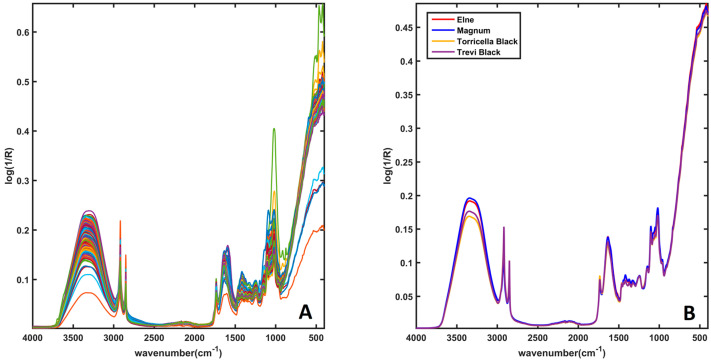
FT-IR spectra. Plot (**A**): spectra of all the analyzed samples. Plot (**B**): mean spectra of samples belonging to the four classes. Legend: Elne—red line; Magnum—blue line; Torricella—yellow line; Trevi—purple line.

Prior to the chemometric analysis, all the spectra were exported from the Spectrum software (PerkinElmer, Waltham, MA, USA) and imported in Matlab (R2015b; The Mathworks, Natick, MA, USA). Figure 1 shows all the 320 spectra associated with the different samples (left plot) and the mean spectrum of each category (right plot). From the plot, it is seen that the diverse types of celery do not present sensibly different absorptions. In general, mean spectra show a broad band centered at around 3300 cm^−1^, attributable to the O−H stretching of water, probably overlapping the vibrations associated with the same bond in sugars and polyphenols, and to the stretching of the N–H bond in proteins. The spectra have peaks also in the range of 2920–2860 cm^−1^, which can be associated with the –CH, –CH_2_, and –CH_3_ stretching vibrations in carbohydrates, sugars, and lipids, whereas those around 1640 cm^−1^ are ascribable to the bending of water and to amide I and II in proteins. Eventually, peaks in the region of 1200–900 cm^−1^ can be attributed to C–H and C–O, bending modes in polysaccharides (mainly pectic and hemicellulosic components of plant cell walls). Other peaks in the range of 1800–1500 cm^−1^ can be associated with the C–C skeletal vibrations (approximately around 1600 cm^−1^) or those associated with the C=C bonds in ketones (α,β-unsaturated). In addition, in the range of 1580 cm^−1^–1540 cm^−1^, vibrations ascribable to C–O bonds in esters can be detected. The C–O stretching vibration in glycosidic C–O–C also provides bands around 1100 cm^−1^ and 1050 cm^−1^ [11,12,13,14,15]. 

### 2.1. Chemometric Analysis

The chemometric analysis consists of a preliminary exploratory investigation made by means of PCA. Eventually, the classification of samples according to the botanical origin has been carried out following two different strategies. The first one, based on a discriminant approach, SPORT-LDA, and the second one, depending on SIMCA.

#### 2.1.1. Principal Component Analysis 

**Figure 2:**
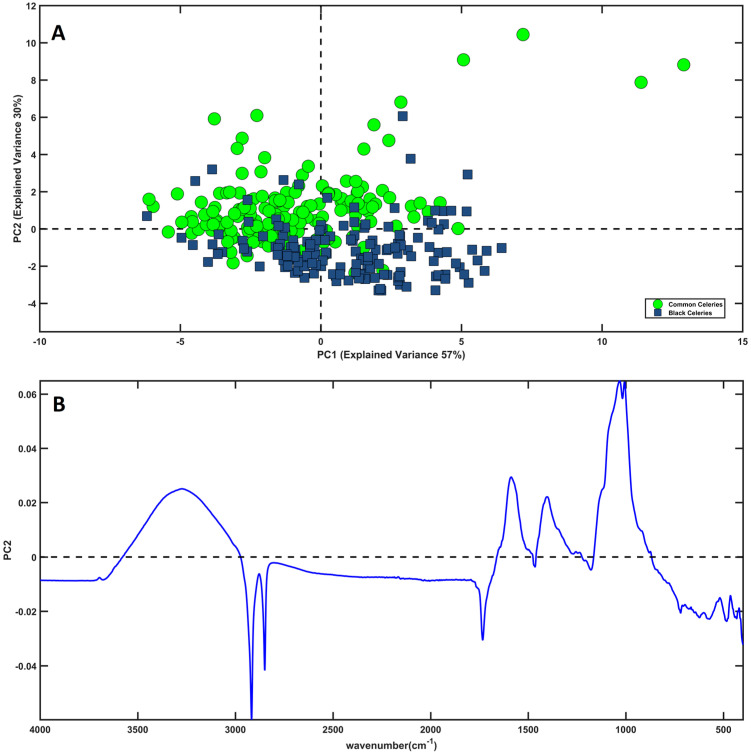
PCA. (**A**) Samples projected onto the space spanned by the first two PCs. Legend: Black Celeries—blue squares; Common Celeries—green dots; (**B**) Loading Plot of PC2.

**Figure A1:**
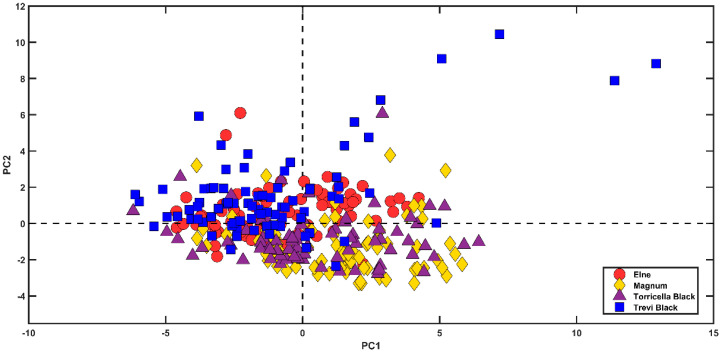
PCA. Samples projected onto the space spanned by the first two PCs. Legend: class Elne—red dots; class Magnum—blue squares; class Torricella Black—yellow diamonds; class Trevi Black—purple triangles.

Initially, a PCA model has been calculated on all the mean-centered IR signals. The investigation of the outcome of the PCA, in particular the diagnostic T2 vs. Q, did not reveal the presence of outliers or suspicious samples. The analysis of the scores plot did not highlight clear clusters related to the four different categories taken into consideration (the PCA-scores plot is shown in Figure A1 in Appendix A). Nevertheless, by restricting the problem to only two classes, i.e., considering black celery samples vs. the common ones, it is possible to recognize some trends. Figure 2A displays the individuals projected onto the space spanned by the first two components; samples of common celery (Elne + Magnum) are represented as green dots, while samples of black celery (Torricella + Trevi) are shown as blue squares. This representation shows that the objects tend to separate along PC2. In fact, except for a certain degree of overlapping, it is possible to note that a great part of common samples presents positive values of PC2, whereas the majority of black celeries fall at negative values of this component. Comparing these observations with the PC2 loading plot (Figure 2B), it is possible to conclude that black celeries are characterized by spectral variables in the range of 2916 cm^−1^–1735 cm^−1^ (negative variables in PC2-loadings), putatively attributable to carbohydrates, water, and sugars. On the opposite side, the common ones are characterized by the broad band centered around 3200 cm^−1^, and by variables in the range of 1600 cm^−1^–1000 cm^−1^, probably associable with a different water content and cell wall structure.

#### 2.1.2. Discriminant Classification of Botanical Varieties

As will be described in Section 3.3, in order to externally validate the classification models, all the available samples were divided into a training (or calibration) and a test (or validation) set. Sample splitting was carried out in order to ensure the representativeness of all classes in the sample space (the reader is addressed to Section 3.3 for more details on the splitting algorithm). The training set was elaborated by means of SPORT-LDA. The tested pretreatments were SNV, first derivative (15 point windows, second order polynomial), and second derivative (15 point windows, third order polynomial). Consequently, the model was calculated on four different predictor blocks. The first modeled block (***X***_1_) was the one incorporating mean-centered data, the second block (***X***_2_) was preprocessed by SNV, and the last ones (***X***_3_ and ***X***_4_) were pretreated by the first and the second derivatives. In order to define the optimal complexity of the model, all the possible combinations of latent variables (LVs) (between 0 and 10, meaning that 0 corresponds to discarding the block) for the different blocks were tested in a seven-fold cross-validation procedure. Eventually, the model providing the lowest classification error in cross-validation (CE_CV_%) was adopted as the predictive one. The chosen calibration model was the one built extracting three, nine, and three LVs from the first three blocks, respectively, which led to a total correct classification rate in cross-validation of 92.2%. The absence of latent variables extracted from ***X***_4_ indicates that the block pretreated by the second derivative has been discarded. The application of the optimal calibration model to the test set led to the correct classification of 90% of the validation samples. This corresponds to the erroneous classification of only 10 test samples out of 100. For class Elne, both sensitivity and specificity were 96%; for class Magnum, they were 80% and 99%; for class Torricella Black, they achieved 96% and 95%, and for class Trevi Black, they were 88% and 97%. The misclassified samples were 1 Elne (predicted as belonging to class Magnum), five objects appertaining to class Magnum (two confused with class Elne, two with class Torricella Black, and one with class Trevi Black), one Torricella Black sample (predicted as belonging to Trevi Black), and three Trevi Black samples (one misclassified with class Elne and two with class Torricella Black). 

**Figure 3:**
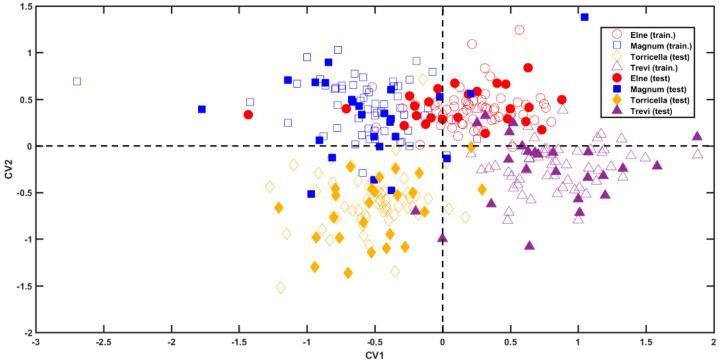
SPORT-LDA analysis. Projection of samples onto the first CVs. Legend: class Elne—red dots; class Magnum—blue squares; class Torricella Black—yellow diamonds; class Trevi Black—purple triangles. Filled and empty symbols represent calibration and validation samples, respectively.

The inspection of the projection of samples onto the first two canonical variates (CVs) (i.e., the directions of maximum separation among classes) [16] shown in Figure 3 reveals a good discrimination of the individuals into the four classes. In particular, the first canonical variate allows the discrimination between class Magnum (blue squares) and class Torricella Black (yellow diamonds), at negative values, from class Elne (red dots) and class Trevi Black (purple triangles), at positive CV1-scores. On the other hand, the second component provides the distinction between the categories coupled along CV1. In fact, class Magnum can be discerned by class Torricella Black because the former falls at positive values of CV2, whereas the latter at negative CV2 scores. Similarly, class Elne (at positive values of the second canonical variate) separates from class Trevi Black (which falls at negative values of the second component) along CV2. 

**Figure 4:**
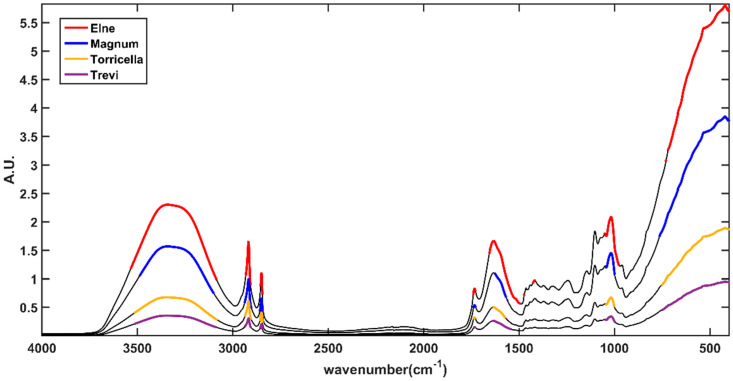
VIP analysis. Black lines represent the average spectra per class. The upmost line is the average spectrum for class Elne. The middle lines are average spectra for class Magnum and class Torricella Peligna Black, respectively. The lowest line is the average spectrum for class Trevi Black. Bold-colored variables are those corresponding to VIP > 1.

SPORT, being a PLS-based method, is compatible with variable importance in the projection (VIP) analysis [17]. This variable ranking approach provides an indication of the variables contributing the most to the solution of the classification problem. Briefly, VIP indices are calculated for each spectral variable, and, customarily, those presenting a VIP index higher than one are considered relevant. The spectral variable characterizing the four categories has been highlighted in the plot in Figure 4. In the figure, black lines represent the mean spectrum of the four classes. Colored variables are those corresponding to VIP indices higher than one. Red variables are associated with class Elne, blue variables with class Magnum, whereas the mustard and purple features refer to class Torricella and class Trevi, respectively. Please note that spectra have been vertically shifted to make all of them visible in the same figure.

Looking at Figure 4, it is possible to observe that the relevant variables belong to the same spectral ranges in each category, indicating that the major differences among them depend on the diverse abundancies of the compounds in the different ecotypes. Celeries are characterized by the spectral variables at 2916 cm^−1^, 2849 cm^−1^, and 1735 cm^−1^, i.e., by the contribution provided by carbohydrates, lipids, and sugars, by the broad band centered at 3276 cm^−1^ and by peaks at 1584 cm^−1^, 1405 cm^−1^, 1029 cm^−1^, and 1007 cm^−1^, probably associable with a different water content and cell wall structure. 

#### 2.1.3. Class-Modeling of Botanical Varieties

As described in Section 3.2, class-modeling approaches are conceived for the classification of individual categories of interest. As a consequence, SIMCA modeling has been circumscribed on the two species of black celery. Unlike SPORT, SIMCA does not allow ensemble preprocessing; therefore, to test different preprocesses, it is necessary to create different models. The tested pretreatments are the same as previously discussed, i.e., bare mean-centering (MC), SNV, first derivative (D1), and second derivative (D2). Eventually, four different SIMCA models (one for each pretreated block) were created within seven-fold cross-validation procedures. 

The results obtained and discussed in terms of efficiency in cross-validation are reported in Table 1. The optimal model was chosen by preferring the maximization of this figure of merit. In the case of equal/similar efficiencies, the model requiring the minimum number of PCs was preferred.

As appreciable, the optimal pre-processings are the first derivative and bare mean-centering for class Torricella Black and class Trevi Black, respectively. 

**Figure 5:**
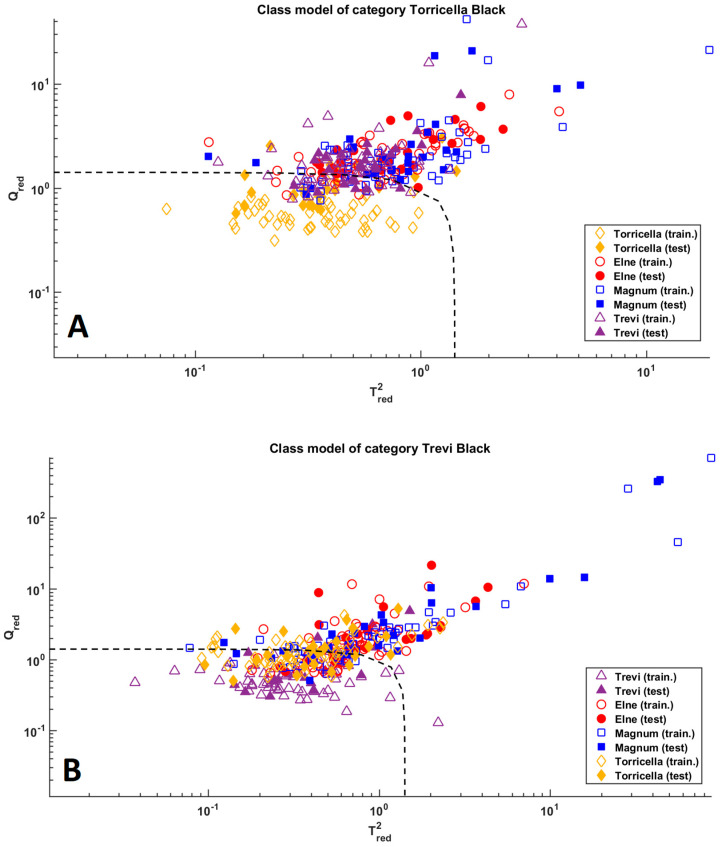
SIMCA analysis. Plot (**A**): model of class Torricella Black; Plot (**B**): model of class Trevi Black. Legend: class Magnum—blue squares; class Torricella Black—yellow diamonds; class Elne—red dots; class Trevi Black—purple triangles. Filled and empty symbols represent calibration and validation samples, respectively. The dashed line delimits the acceptance region.

The application of these models to the test set led to 68% of sensitivity and 85.3% of specificity for class Torricella Black and to 84% of sensitivity and 68.0% of specificity for class Trevi Black. These results are also graphically shown in Figure 5. From the figure, it is clear that, concerning class Torricella Black (top subplot in Figure 5), the model has a high specificity (i.e., it properly performs at rejecting samples that do not belong to the modeled class), but low sensitivity. On the other hand, the plot associated with the model of class Trevi Black shows the opposite trend encountered in this category, i.e., a high sensitivity (68% of samples appertaining to the modeled class are correctly accepted) is accompanied by a low specificity, resulting in a model which (erroneously) accepts too many individuals. 

## 3. Materials and Methods

### 3.1. Samples

Four varieties of celery (*Apium graveolens* L.) were investigated: “Elne”, “Magnum”, Torricella Peligna Black, and Trevi Black. The first two types are common varieties of celery, easily available in (super)markets in Italy, while the latter two are niche types of black celery, mainly grown in specific areas of central Italy (Abruzzo and Umbria). Black celery from Trevi is protected by the Slow Food Presidia, whereas black celery from Torricella Peligna is in the process of being registered in the regional Abruzzo registry of plant biodiversity, conceived for in situ or ex situ conservation of varieties at extinction risk. 

Several plants of these four types of celery have been grown in an experimental field located in Torricella Peligna (Abruzzo). The seeds were planted on the 12th of May 2021 and harvested on the 1st of December 2021. It has to be noted that the plants of the different types of celery involved in the study were all grown in experimental fields close to the territory of Majella National Park, consequently, under the same soil and climatic conditions. 

Eventually, petioles (including leaves) were collected from both the outside and the inside of the stalks and stored in a freezer at −18 °C. Prior to the analysis, each single leaf sample was left to thaw, and dabbed with absorbent paper. The leaves that showed imperfections, spots, or breakage of various kinds were discarded. Before proceeding with the analysis, a visual investigation was also carried out to ensure that there was no soil or mud on the leaves. Eventually, samples were analyzed by ATR-FT-IR. For each type of celery, 80 leaves (from different plants) were analyzed. The analysis took place as described in Section 3.2.

### 3.2. Collection of ATR-FT-IR Spectra

The analysis was carried out using an FT-IR spectrometer (PerkinElmer Spectrum Two™-PerkinElmer, Waltham, MA, USA). For each measurement, one single leaf was placed on the appropriate support of the instrument. The leaf was then pressed on the single-bounce diamond (PerkinElmer Universal Attenuated Total Reflectance (uATR) diamond crystal), checking that the pressure was approximately the same for each collected spectrum.

The spectral range explored was 4000 cm^−1^–400 cm^−1^ (4 cm^−1^ resolution), and 16 scans were collected for each individual sample. The entire data set consisted of 320 spectra (80 for each type of celery), resulting in a data matrix **X** of dimensions 320 × 3601. Between one leaf and another, the diamond was cleaned with a soft tissue moistened with methanol. After a few minutes of waiting, a sample-free spectrum was collected to check for the absence of methanol residues.

### 3.3. Chemometric Model-Building and Validation

The aim of the present work is to classify celery samples according to their type. In order to achieve this goal, discriminant and class-modeling methods were used. These two different strategies were chosen in order to evaluate how approaches of different natures would behave on the samples under examination. 

Initially, all the available data were investigated using an explorative method in order to unveil trends within them. For this purpose, the principal component analysis (PCA) [18] was exploited, which allows the bilinear decomposition of the data matrix **X** according to Equation (1):

**X = TP^t^ + E** (1)

where **T** and **P** are the scores and the loadings matrix, respectively, and **E** contains the residuals. Briefly, this represents a compression of information into an extremely reduced number of latent variables (the *PCs*), which allows the creation of plots (scores and loadings plots) aimed at interpreting the system.

Subsequently, after the exploratory analysis, and after the verification that there are no outliers or suspicious samples, it is possible to proceed with the creation of the classification models.

The discriminant classifier used is SPORT-LDA [19], an approach derived from Sequential and Orthogonalized Partial Least Squares Linear Discriminant Analysis (SOPLS-LDA) [20]. SPORT is an ensemble preprocessing method that allows extracting information from pre-treated data blocks; eventually, its combination with linear discriminant analysis (LDA) [21] endorses classification. The pre-processing methods used were as follows: Standard Normal Variate (SNV) [22], the first (D1) and the second (D2) derivatives calculated employing the Savitzky–Golay approach [23], using 15 point windows, and the second and the third order polynomial, respectively. In theory, SPORT allows to model any number of predictor blocks. In this case, the investigation was restricted to the methods that seemed the most appropriate for the data under investigation. Among the various discriminant methods available in the literature, SPORT-LDA was chosen because it has several benefits. Among the main ones is its ability to remove the redundancies among the analyzed data blocks (this derives from the fact that, before extracting information from the different data blocks, they are orthogonalized with respect to the information extracted from the already modeled ones). For more details on the SPORT algorithm, the reader is addressed to the literature [19,24,25].

The second exploited strategy is based on the SIMCA [26], which is one of the most widely used class-modeling methods. Given its nature, it is particularly suitable for handling the so-called asymmetric classification problems, i.e., all those situations where the interest lies in a particular class. In fact, this approach is based on the individual modeling of categories. Very briefly, in order to build a SIMCA model, the first step is to calculate a PCA model on the class of interest. Then, the distance from this model and all the investigated objects is estimated. According to this entity, an individual will be accepted by the model (and predicted as belonging to the modeled class) or rejected (and not considered part of the category of interest). 

In the present work, the SIMCA-modeled categories are those associated with black celeries. Consequently, in Section 2, only the models associated with class Torricella Peligna Black, and class Trevi Black will be discussed. For the algorithm of SIMCA, and details about its application, the reader can refer to [26,27]. Contrary to SPORT-LDA, where the outcome of the analysis is generally provided in terms of total predictive accuracy/classification error, in SIMCA, the results are discussed in terms of sensitivity, specificity, and efficiency. The first entity refers to the percentage of samples correctly accepted by the class model; the second represents the percentage of objects properly rejected by the class model, while the efficiency is the geometric average between the two. This last figure of merit is the one that is investigated in cross-validation in order to decide which calibration model is more suitable for the data under examination.

In order to externally validate the classification models, all the available samples were divided into a training (or calibration) and a test (or validation) set. To ensure the representativeness of the distribution of samples in the four classes, the Duplex algorithm [28] was individually applied to the spectra belonging to every single category. Additionally, 25 objects per class were selected to be part of the test set; consequently, the calibration set was made of 220 samples (55 per class), and the validation set of 100 spectra.

## 4. Conclusions

The present study investigated the characteristics of four ecotypes of Italian celery. To ensure that the observed differences mainly derive from the different types of celery, the analyzed plants were all grown in the same experimental field (under the same growing conditions). The application of both SPORT and SIMCA to the IR signals collected on the 320 samples revealed significant differences among the diverse types of celery. Both classifiers achieved good accuracies in prediction on the test set; the SPORT-based strategy is the one that allowed obtaining the best results from the predictive point of view. In fact, it led to the correct classification of 90% of the validation samples. In general, it is possible to conclude that Elne, Magnum, Torricella Black, and Trevi Black celeries have different characteristics which are distinguishable by IR analysis combined with SPORT. This is an interesting outcome, supporting the fact that the local varieties of celeries grown in Torricella Peligna and in Trevi have peculiarities that distinguish them from common celeries.

## Figures and Tables

**Table 1:** SIMCA results associated with the modeling of class Torricella Black and class Trevi Black.

**Class Torricella Black**		
**Pretreatment**	**PCs**	**Efficiency (%CV)**
MC	10	74.5
SNV	11	70.9
D1	9	80.0
D2	10	80.0
**Class Trevi Black**		
**Pretreatment**	**PCs**	**Efficiency (%CV)**
MC	8	80.0
SNV	11	69.1
D1	13	70.9
D2	12	70.9

## Data Availability

The data presented in this study are available on request from the corresponding author.

## Appendix A

A PCA model was calculated for all samples, taking care of the four different categories. No clear clusters can be noticed in the PCA-scores plot (Figure A1).
